# Core components, concepts and strategies for parasitic and vector-borne disease elimination with a focus on schistosomiasis: A landscape analysis

**DOI:** 10.1371/journal.pntd.0008837

**Published:** 2020-10-30

**Authors:** Nora Monnier, Tanja Barth-Jaeggi, Stefanie Knopp, Peter Steinmann

**Affiliations:** 1 Swiss Tropical and Public Health Institute, Basel, Switzerland; 2 University of Basel, Basel, Switzerland; University of the District of Columbia, George Washington University School of Medicine and Health Sciences, UNITED STATES

## Abstract

Efforts to control and eliminate human schistosomiasis have accelerated over the past decade. In a number of endemic countries and settings, interruption of schistosome transmission has been achieved. In others, *Schistosoma* infections continue to challenge program managers at different levels, from the complexity of the transmission cycle, over limited treatment options and lack of field-friendly accurate diagnostics, to controversy around adequate intervention strategies. We conducted a landscape analysis on parasitic and vector-borne disease elimination approaches with the aim to identify evidence-based strategies, core components and key concepts for achieving and sustaining schistosomiasis control and for progressing elimination efforts towards interruption of transmission in sub-Saharan Africa. A total of 118 relevant publications were identified from Web of Science, Pubmed and the grey literature and reviewed for their content. In addition, we conducted in-depth interviews with 23 epidemiologists, program managers, policymakers, donors and field researchers. Available evidence emphasizes the need for comprehensive, multipronged and long-term strategies consisting of multiple complementary interventions that must be sustained over time by political commitment and adequate funding in order to reach interruption of transmission. Based on the findings of this landscape analysis, we propose a comprehensive set of intervention strategies for schistosomiasis control and elimination. Before deployment, the proposed interventions will require review, evaluation and validation in the frame of an expert consultation as a step towards adaptation to specific contexts, conditions and settings. Field testing to ensure local relevance and effectiveness is paramount given the diversity of socio-ecological and epidemiological contexts.

## Author summary

This landscape analysis explored successful concepts, approaches and interventions of past and ongoing parasitic and vector-borne disease elimination efforts and programs with regard to relevance for progress in the elimination of human schistosome infections. Schistosomiasis is a disabling, water borne parasitic disease of public health concern with an estimated 250 million people infected worldwide. The long-term morbidity of this neglected tropical disease significantly impacts growth, cognition and socioeconomic development at all ages. Despite increased global efforts to control morbidity and advance elimination, challenges in view of the complex life cycle which involves freshwater sources, intermediate snail hosts and humans, remain. This calls for targeted interventions and concerted programs. According to the evidence from the literature and as proposed by a wide range of key informants, comprehensive, multipronged and long-term strategies supported by strong political commitment and adequate funding are required in order to achieve and sustain the set goals. Based on the findings, we propose here a comprehensive set of intervention strategies for schistosomiasis control and elimination for review and evaluation to inform implementation research needs and elimination program design.

## Introduction

Schistosomiasis is a neglected tropical disease (NTD) with considerable impact on global health [1]. Five different *Schistosoma* (blood fluke) species have been described to affect humans, all of which depend on either aquatic or amphibious snails as intermediate hosts. Transmission to the human host occurs during exposure to water bodies infested with cercariae (schistosome larvae). Infection can result in acute and chronic disease manifestations in all age groups [2, 3].

Historically, endemic countries mainly relied on snail control and environmental modification in their efforts to control schistosome transmission, since treatment options were limited due to concerns about the safety of then available drugs [4]. Since the mid-1980s, praziquantel allows safe treatment [5]. As a consequence of the introduction of praziquantel, efforts in endemic countries were refocused on controlling the morbidity due to schistosomiasis, mainly through periodic mass drug administration (MDA) of praziquantel to the at-risk population without prior diagnosis, a concept also known as “preventive chemotherapy”[6]. A paradigm shift occurred in 2012 when the declared goal of the World Health Organization (WHO) was revised from morbidity control by 2020 to eliminating schistosomiasis as a public health problem, and to interrupt transmission in selected areas by 2025 [7]. Control of morbidity was suggested to be achieved through large-scale administration of praziquantel to populations at risk using defined thresholds of prevalence as criteria for selecting the appropriate interval of re-treatment. It was also emphasized that for elimination as a public health problem and interruption of transmission, preventive chemotherapy intervals needed to be adjusted and intensified, and complementary public health interventions were strongly recommended if not deemed essential [7]. In 2020, the WHO Roadmap for NTD control 2021–2030 was published. It measures progress in schistosomiasis control in terms of number of countries where elimination as a public health problem (<1% heavy intensity infections) has been validated. It also states that continued actions are required to maintain these achievements and to further advance elimination through interruption of transmission in defined geographical areas [8].

In several countries, most notably Japan, the goal of breaking schistosome transmission has long been achieved [4, 9]. Nationwide efforts in P.R. China and several other countries including in the Caribbean, have succeeded in eliminating schistosomiasis as a public health problem through integrated intervention efforts [9–11]. Multipronged strategies tailored to the local microepidemiology are needed to interrupt transmission. This was again highlighted by recent studies from the Zanzibar islands of the United Republic of Tanzania where ten rounds of biannual MDA implemented over 5 years were not sufficient to interrupt *Schistosoma haematobium* transmission [12, 13]. This is in contrast to estimates from mathematical models suggesting MDA alone could result in transmission interruption if maintained over sufficient time at moderate to high coverage of school-aged children and other key community groups [14, 15].

Despite considerable progress and scaling-up of interventions over the past decades, a high burden of disease due to schistosomiasis persists in sub-Saharan Africa. Sustained transmission has been attributed to factors related to socio-economic development, weak health systems, a lack of country ownership and behavioral aspects in regards to dependency on open water sources and livelihoods [16–18]. In addition to these endogenous factors, limitations in field-applicable and affordable, highly sensitive and specific diagnostic tools, scalable biological vector control methods and a lack of novel drugs present ongoing bottlenecks and threaten global elimination efforts [11].

To improve the effectiveness of interventions that can contribute to realizing the goals set by WHO, it is necessary to review current intervention strategies, tools and guidance for treatment and control, and to identify innovations to overcome past and ongoing program failures, compliance and acceptance issues [19]. Consequently, we conducted a landscape analysis on interventions and strategies for parasitic and vector-borne disease elimination including a literature review and key informant interviews. The objective was to identify evidence-based components, concepts and intervention strategies that can help to advance schistosomiasis control towards elimination as a public health problem and interruption of *Schistosoma* spp. transmission across a variety of settings, with a focus on sub-Saharan Africa.

## Methods

### Literature review

The scientific literature databases PubMed and Web of Science were explored using the string search ((elimination OR eradication) AND vector-borne) OR ((elimination OR eradication) AND parasit*). No restrictions were applied with regard to study location, date of publication, article type, or language. Hits were imported into the online software “Covidence” for title and abstract screening. Two independent reviewers then stratified potentially relevant references into high- and low priority based on the title. Important criteria for the classification as high-priority articles were i) being a review or opinion piece on innovative intervention strategies; or ii) the description of comprehensive control or elimination programs. Overviews over research needs and program updates were regarded as lower priority articles. High-priority references underwent a detailed full text screening by two independent reviewers using a data extraction matrix in Microsoft Excel to capture relevant information for further assessment and final inclusion. Eligibility disagreements were resolved by discussion and reference to a third reviewer. Final inclusion of an article was based on the description of interventions and concepts relevant for vector-borne disease elimination. Findings were summarized in an excel table (see S1 Table) and stratified into 6 thematic groupings, namely i) treatment, ii) vector control, iii) water, sanitation and hygiene (WASH), iv) information, education and communication (IEC), v) surveillance and information systems, and vi) conceptual design of program implementation (see S2 Table). Complementary evidence was obtained from the grey literature underlining the findings from the peer-reviewed literature.

### Key informant interviews (KII)

In-depth interviews were conducted with key informants with first-hand knowledge on parasitic and vector-borne diseases, disease elimination and public health programs, field researchers, program managers, policymakers and donors. The interviews were conducted in person, via skype or by telephone. Sampling was purposive and informants were first identified through personal and professional networks. In addition, a snowball sampling technique was used during interviews for experts to nominate additional relevant contacts for recruitment. Information on the background and life cycle of schistosomiasis was provided before the interview and in addition, a semi-structured questionnaire guided by a specific scenario (schistosomiasis elimination efforts in Zanzibar: a setting with a long history of control and elimination activities, with a now very low prevalence and predominantly light-intensity infections) was available upon request. A semi-structured interview guide was developed by the authors and sent to the key informants (see S1 Appendix). After verbal consent of the interviewees had been obtained, interviews were audio-recorded and transcribed for further analysis by two researchers. The interview transcripts were analyzed by summarizing them in a data extraction matrix modelled on the example used for the literature review (i.e., stratified into 6 thematic groupings, namely i) treatment, ii) vector control, iii) water, sanitation and hygiene (WASH), iv) information, education and communication (IEC), v) surveillance and information systems, and vi) conceptual design of program implementation, with a view to identify key topics and components (see S3 Table).

## Results

### Literature review and key informant interviews

**Fig 1:**
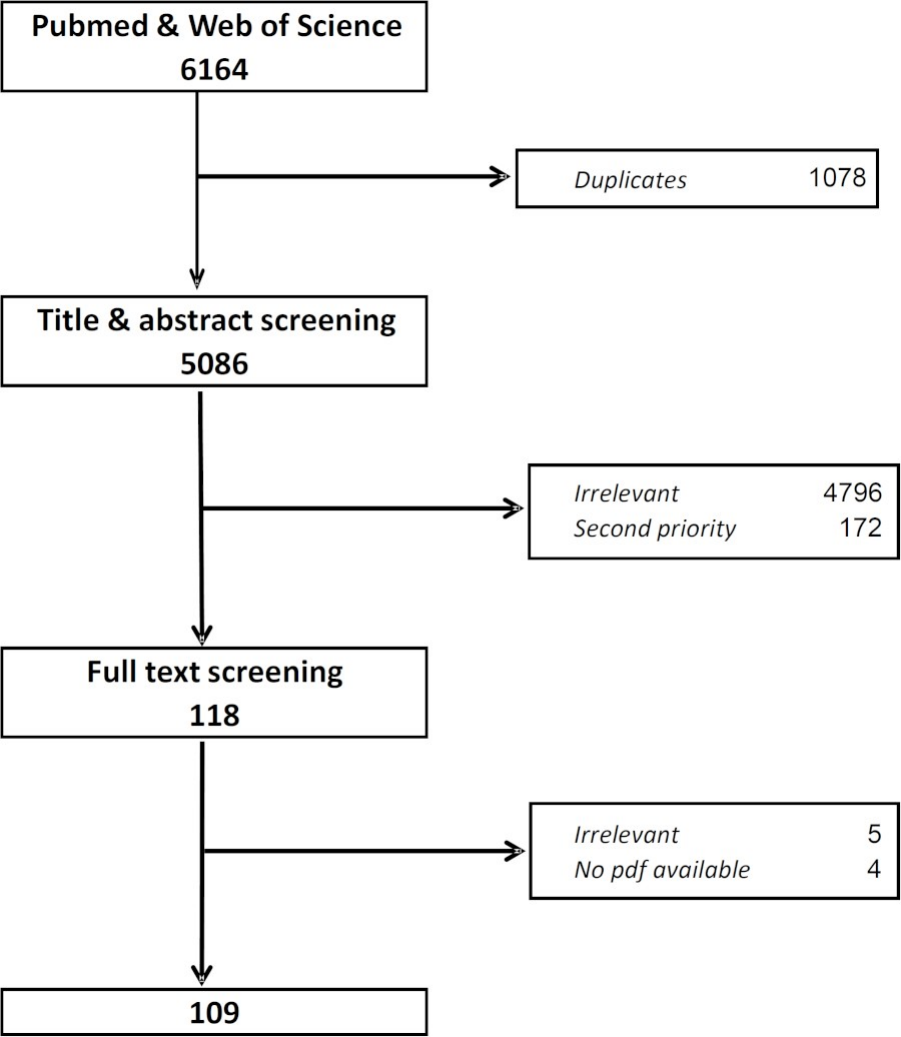
Flow-chart of article selection for inclusion in the literature review on vector-borne disease elimination strategies.

The final literature database search was conducted on 12 March 2019. As shown in Fig 1, applying the search algorithm in the two databases yielded a total of 5’086 hits. After exclusion of duplicates and irrelevant articles, 290 titles were retained and subsequently stratified into high- and low-priority references based on the above-mentioned criteria. A total of 118 articles were classified as high-priority and selected for full-text screening. Among them, four full texts were not obtainable from online archives and through the scientific library services. Among the remaining 114 articles, five were excluded because upon review of the full text it appeared they did not describe interventions of potential relevance for parasitic and vector-borne disease elimination. The final list included 109 articles that underwent content analysis.

Key informant interviews were conducted with 23 participants with a background in parasitic and vector-borne diseases and elimination. Included were representatives of international organizations, academic and implementation research experts, program managers, policymakers and donors from 6 continents including endemic regions in Africa, Asia, and the Americas, between 18.03.2019 and 20.06.2019.

The combined findings from the literature review and key informant interviews are presented below, stratified by the 6 thematic groupings (i.e., i) treatment, ii) vector control, iii) water, sanitation and hygiene (WASH), iv) information, education and communication (IEC), v) surveillance and information systems, and vi) conceptual design of program implementation).

#### Treatment

Periodic praziquantel-based MDA to school-aged children is the mainstay of most current schistosomiasis control programs worldwide. The key informants highlighted a series of strategies, which in their opinion could improve MDA treatment coverage and compliance with a view to cover all at-risk populations. Strategies ranged from large-scale risk assessments over focused mapping of risk groups to inform targeted and selective treatment efforts, to tailored and adaptive strategies which address residual high transmission foci amid generally low prevalence populations. Tailoring interventions and targeted treatment strategies entails a need for clear guidance on which populations and individuals at risk to screen, and when to implement focal or large-scale mass drug administration, or, potentially, test-and-treat approaches.

Targeted treatment approaches were suggested to avoid unnecessary treatment of healthy people (overtreatment) and to enhance acceptance, compliance and coverage in the population. In consideration of a potential risk of drug resistance and to improve clearance of parasites, it was referred to studies indicating that varying the frequency of treatment and a combination of praziquantel with artemether might offer alternatives to the current standard of periodic treatment with praziquantel for schistosomiasis. Artemether, a derivative of artemisinin and key component of artemisinin-based combination therapy (ACT) against malaria, has been shown to kill immature schistosome stages that are not susceptible to praziquantel. It was suggested to (re)-considered using artemether, but only in settings where malaria is not endemic to avoid the promotion of resistance development to antimalarials.

To avoid the formation of persisting “reservoirs” of infected individuals maintaining transmission, it was highlighted that treatment efforts should target the entire at-risk population at high coverage. To this end, key informants emphasized that availability and access to treatment beyond school-based programs need to be strengthened. Thus, for targeting schistosomiasis, praziquantel must be made available through routine health services and drug distribution be integrated into existing platforms such as child health days.

The views of key informants were confirmed by the findings from the literature review, where countries including Oman, Venezuela, Lao PDR, Cambodia and P.R. China reported the successful combination of targeted schistosomiasis screening and selective/targeted treatment with the aim to avoid the treatment of non-infected individuals and to increase compliance for treatment against *Schistosoma* infections in endemic areas [20–29]. The value for decision-making of tailored interventions to target asymptomatic carriers contributing to transmission through community-wide mass drug treatment and active case finding, in addition to mapping spatial and temporal transmission dynamics, has been established for malaria [30–34].

In terms of praziquantel alternatives, limited experience with combining artemether and praziquantel for targeting both the juvenile schistosomula and adult parasites has been published [35, 36].

#### Vector control

Many successful efforts to eliminate schistosomiasis have included snail control and environmental modification as key interventions [4]. Both the interviewed experts and the literature emphasized the importance of vector control when considering disease elimination targets. Vector control has been identified as a key factor for the successful elimination of schistosomiasis in Japan, and national programs in P.R. China and Puerto Rico relied in an important way on snail control supported by WASH and health education [29, 37–42]. Similarly, vector control has been instrumental for the control and elimination of other parasitic and vector-borne diseases such as malaria, dengue, onchocerciasis and Guinea worm [43–57].

In summary, as expressed by key informants and described in the literature, snail control is a valuable component of schistosomiasis control efforts, and mapping infested water sources to inform small-scale and focal mollusciciding (by hand or through GPS-guided drones) is considered essential where biological methods are not available [4, 11, 58]. Better training and community engagement were recommended by the key informants to reduce environmental and monetary costs and to increase sustainability and acceptance of vector control activities in the face of toxic molluscicides. The guide on field use of molluscicides for schistosomiasis control issued by WHO serves as an operational manual for programme managers on snail control and informs decision-making [59]. Engaging and training communities in vector control activities to foster ownership and sustainability have proven feasible in community-based integrated vector control programs for dengue, malaria and lymphatic filariasis (LF) [43, 46, 60–63]. Reported mainly in the Chinese literature, large-scale interventions such as changes in land use and farming practices, civil engineering and water resource development projects highlight the need for engagement with multiple sectors for investments, coordination and risk assessment to ensure sustainability and impact. Most recent published experience in the domains of environmental, agricultural and ecological modifications is concentrated in P.R. China and relates to *S*. *japonicum* in humans, animals and snails [38–42]. Mollusciciding has not been widely implemented in sub-Saharan Africa, and biological control approaches and trials are limited to specific settings. However, gene-drive technologies, cost-effectiveness analyses, modelling and innovations to assess snail abundance for targeted snail population management offer promising future pathways [4, 64, 65].

#### Water, sanitation and hygiene (WASH)

WASH infrastructure and behavior are key for the control of water-borne and water-related diseases including NTDs, and are critical for schistosomiasis control and elimination efforts [66]. The WHO, in 2019, published a “how-to”-guide that offers practical advice for NTD programs interested in establishing ties with the WASH sector [67].

As stressed by several interviewed key informants and documented in the literature, safe water and improved sanitation installations must be locally and culturally appropriate and tailored to environmental conditions and the socio-economic context [68]. Key informants emphasized that community involvement in the planning and implementation of interventions is crucial to ensure uptake, acceptance and sustainability of WASH interventions. Successful examples of comprehensive community-led MDA combined with community-led WASH (CL-WASH) interventions for schistosomiasis control in Cambodia and Lao PDR have been published [24, 38].

#### Information, education and communication (IEC)

Effective and well-executed IEC and behavior change (BC) interventions are imperative for disease control and elimination in terms of acceptance by the target population and sustainability of interventions, but have long been neglected due to difficult standardization and the scarcity of proven models [69–72]. Interviewed key informants highlighted that a focus should be placed on raising awareness for the disease and interventions in the target population and among relevant stakeholders to facilitate diagnosis, treatment and surveillance activities. Several key informants pointed out that early childhood education has proven effective in adapting and improving WASH behavior and is applied for trachoma and soil-transmitted helminth (STH) control and prevention through habit formation. Community participation was identified by key informants and in the literature as crucial to maximize acceptance, gain a sense of ownership and empowerment and achieve sustainability of interventions and activities including vector control, WASH, surveillance, treatment, health education and behavior change communication in programmes targeting malaria, dengue, schistosomiasis, Guinea worm and other parasitic and vector-borne diseases [11, 24, 43, 46, 53, 60–62, 72–76]. Caregivers, social groups, religious or community leaders, teachers and health staff should be engaged in control activities, intervention planning and design of tools [77].

Health education and health promotion have been documented to play an integral role in national schistosomiasis control program efforts in P.R. China [69, 71] and Brazil [72]. Comprehensive and well-executed activities around disease awareness, knowledge and risk mitigation improved intervention acceptance and compliance rates, and reduced water exposure for parasitic and vector-borne diseases [70, 71, 75, 78]. Toolkits, as developed during the Zanzibar Elimination of Schistosomiasis Transmission (ZEST) program and adapted to local context offer guidance and educational material for engaging with pupils and communities in activities related to prevention, control and treatment (https://www.eliminateschisto.org/resources/teacher-toolkits-for-schistosomiasis). Community participation in sensitization and awareness raising also increased acceptance of preventative measures (vector control) for malaria control in Rwanda [53].

#### Surveillance and information systems

The essential basis for planning interventions for parasitic and vector-borne disease elimination is accurate knowledge on the spatial distribution of infected individuals and transmission sites to identify implementation gaps and inform interventions needs. Interviewed key informants stressed that mapping (including spatio-temporal, precision, fine-scale and micro-mapping) of human and animal sources of infection with pathogenic organisms and associated factors including vectors, transmission sites and relevant human behavior are key for planning targeted interventions. They also indicated that Geographic information system (GIS), remote sensing (RS) and other GeoHealth technologies are well developed for schistosomiasis control and that network modeling and tracking human mobility can inform interventions on a larger scale and across regions.

High-quality data and rigorous surveillance response systems are critical for an effective control and elimination program as was mentioned with particular reference to Guinea worm eradication efforts. It was pointed out that this requires early and sustained investments in a comprehensive surveillance system including routine (periodic, integrated into the primary health system) and active surveillance (i.e. active case detection, tracing and investigations). Key informants stressed that surveillance activities should be performed at all levels (community, district, national, regional) and are an essential component in the “endgame” for monitoring residual human infections as well as for detecting emerging zoonotic reservoirs. Surveillance must be maintained throughout the different stages of elimination programs, as presented in the Transmission Assessment Survey (TAS) framework for LF [79, 80]. As recommended by key informants and in the literature, surveillance should involve community members and primary health care systems [48, 52]. Surveillance strategies call for integration across several diseases to maximize cost-effectiveness [81].

Concepts for schistosomiasis surveillance systems designed for elimination programs, including early warning systems [25, 58, 82–85], mapping of hot spots and modelling for targeted approaches [58, 83, 86] are covered in several reviewed articles. Challenges due to the lack of sensitive diagnostic tools for monitoring and validating stages of elimination in regards to low-prevalence settings and low intensity infections were discussed in articles pertaining to schistosomiasis control programs in Morocco [87], Venezuela [21, 23], Oman [20] and P.R. China [28]. High-quality surveillance and mapping requires sensitive tools for assessing the epidemiology and detecting re-emergence in humans, animals and vectors. Relevant diagnostic tools for schistosomiasis surveillance include molecular diagnostic such as quantitative PCR (qPCR) and loop-mediated isothermal amplification techniques (LAMP) and intermediate host surveillance methods or the detection of environmental DNA, some of which are currently not widely available [88–90].

Several identified articles discussed strategies for surveillance and response systems to achieve elimination of malaria. For example, P.R. China’s comprehensive 1-3-7-strategy for malaria elimination [48, 50] monitors and guides elimination activities including case reporting (e.g. cell-phone based alert system) within 1 day through the disease surveillance information report management system. As outlined in China’s national malaria elimination strategy, case investigation and laboratory confirmation occur ideally within 3 days while response actions including preventive chemotherapy to individuals or populations at risk, community health education, and vector control in at-risk houses are implemented within 7 days. Nearing elimination in many settings, LF control is also facing increased surveillance needs. In response, specific protocols have been developed that offer stepwise guidance on targeted responses for different stages of elimination and highlight the importance of adequate post-intervention surveillance tools to detect re-emergence of infections [79, 80].

#### Conceptual design of program implementation

The interviewed key informants stressed that interruption of schistosome transmission is only feasible with an integrated, multipronged, cross-sectoral and locally adapted strategy. The basis for such comprehensive interventions is collaboration including joint planning, central coordination of different sectors and effective community engagement. Regular progress reviews and strategy updates are essential. Barriers around the establishment of inter-sectoral collaborations are manifold despite common goals, and long-term political commitment to schistosomiasis elimination is paramount to ensure focus and adequate funding [91]. Key informants emphasized that the lack of technical and management capacity at national and local level must be addressed through capacity building and health systems strengthening through training of program managers, health and laboratory staff and community members.

Most of the identified articles that focus on strategies for implementation of interventions refer to the Chinese schistosomiasis control programme. They describe a combination of tools and comprehensive strategies [11, 20, 21, 23, 36, 90, 92–94] implemented through collaborations between multiple sectors but under central coordination [26, 29, 39, 95, 96]. This approach has been identified as important to reduce schistosomiasis japonica levels across P.R. China [22, 26, 27, 29, 39, 42, 95–97]. The value of integrated One Health approaches has been described in the context of *Schistosoma mekongi* elimination efforts in Lao PDR and Cambodia [24]. Other Asian countries and Puerto Rico reported similar experiences [37, 38, 90].

The need for multipronged elimination strategies has also been highlighted for other parasitic and vector-borne diseases including malaria [32, 44, 49, 98–100], dengue [43, 46, 56, 60], onchocerciasis [57] and Guinea worm [45, 52, 54]. Examples of integrated, inter-programmatic and cross-sectoral initiatives have been outlined in detail for prevention, control and elimination of NTDs in the Americas [63]. Community-based integrated strategies were also used for polio and measles elimination [101]. Integration across MDA programs and diseases in regards to concurrent administration of multiple drugs exist for schistosomiasis, soil-transmitted helminthiasis and LF [63, 102]. Beyond exploiting synergies from distributing drug combinations, malaria and LF control programs have paired MDA with vector control activities to ensure sustainability of transmission suppression [47].

### Challenges, gaps and needs for achieving interruption of schistosome transmission

The following challenges, gaps and needs were identified from the literature or highlighted by the interviewed key informants.

#### Treatment

There is little evidence in the peer-reviewed literature on schistosomiasis treatment strategies targeting populations beyond school-aged children, despite recommendations by WHO to offer regular treatment to all at-risk populations. However, epidemiological considerations and modelling results suggest that a focus on MDA for schoolchildren risks ignoring a potentially substantial reservoir (and morbidity burden) [14, 36, 75, 92, 93, 103, 104]. Risk factors related to occupation, behavior, and proximity to, as well as dependency on, open water sources have long been identified as challenges to elimination efforts, further underscoring the need to treat also other at-risk populations [105, 106] at high coverage [92, 93, 103].

Mass drug administration may also lead to treatment fatigue, non-compliance and therefore low coverage [36, 107, 108]. Investment in research and development for new anti-schistosomal drugs is needed in view of concerns related to emerging drug resistance and availability of a pediatric formulation of praziquantel suitable for administration to infants [109, 110]. The factors above as well as the opinions of key informants, call for mapping of populations or individuals at risk to facilitate a targeted approach, following clear and comprehensive treatment guidelines, and supported by robust monitoring and evaluation (M&E) adapted to stages in elimination. Guidance on the design of effective test and treat strategies is currently lacking as are affordable, field-ready point-of-care tests, especially for *S*. *haematobium*, that are needed to facilitate test-and-treat options.

#### Vector control

Since the discovery of praziquantel in the 1980s snail control has been increasingly neglected as a key component for many schistosomiasis control and elimination strategies. The toxicity, costs and operational issues associated with chemical mollusciciding also present an ongoing challenge in the face of elusive alternatives. The main issues related to snail control include the contamination of water and food sources resulting in niclosamide being banned in certain locations such as in rice fields in the Philippines. The inaccessibility of certain snail breeding sites, e.g. in the Mekong river, also presents challenges [38]. In March 2020, the WHO has urged member states to include snail control as an integral and crucial component for reaching elimination of schistosomiasis [111]. Chemical mollusciciding, unlike former Dichlorodiphenyldichloroethane (DDT) spraying or long-lasting insecticidal bed nets and indoor residual spraying (LLIN/IRS) for malaria control, is neither sustainable and cost-effective in the long-term, nor feasible for application at large scale [29].

Several articles identified a need for innovative biological snail control and suggested microbial pathogens for the control of *B*. *glabrata* [112] and snail “diagnostics” surveillance for risk mapping with LAMP [88].

#### Information, education and communication (IEC)

Many countries’ efforts and success in eliminating schistosomiasis include strategies relying, among else, on comprehensive health education and communication activities. Well executed, locally appropriate and culturally sensitive IEC can improve community acceptance and participation in all dimensions of a control or elimination program considerably, and facilitates behavioral change in the population. Many current health educational interventions are ineffective and do not lead to behavior change due to a lack of local relevance, effective models, implementation guidance and skilled personnel [113]. IEC and behavior change activities require a time consuming process, and limited interest among control programs and donors so far resulted in little investment in innovative IEC and BC interventions [77].

#### Water, sanitation and hygiene (WASH)

WASH interventions essentially aim at preventing contamination of fresh water bodies with urine and stool while reducing human exposure to unsafe water sources. Successful WASH programs include sectors such as engineering, water authorities, non-governmental organizations (NGOs) and environmental health agencies for the installation of sanitary and hygiene infrastructure, making safe water accessible, and providing health education. Hardware-based solutions have shown limited impact on the local epidemiology of schistosomiasis unless they are accompanied by sustained changes in water contact behaviors [12, 68]. Monitoring of integrated WASH/NTD implementation and generating evidence on the impact of strategies need to be enforced in view of long term investment and sustainability [66].

#### Surveillance and information systems

The paramount importance of understanding local disease and transmission patterns, context and structures using a systems epidemiology approach is described by Krauth et al. [114]. Mapping and surveillance are essential activities for planning and managing schistosomiasis at different stages of elimination efforts [76]. Interviewed key informants highlighted that ecological and human surveillance systems needed to be established to detect transmission-related factors (e.g. zoonotic hybrid schistosomes, animal and human reservoirs, transmission foci) and inform responses. A lack of point-of-care, low-cost and sensitive diagnostic tools contributes to the challenge of defining and understanding the epidemiology of low-intensity and asymptomatic infections and the relevance of animal reservoirs. In addition, cases with low- or asymptomatic clinical presentation challenge traditional case detection approaches and suggest a need for better guidance including test-and-treat algorithms. Last, adequate resources and capacity at local and national levels of endemic countries are required to appropriately interpret data and design timely response activities.

#### Conceptual design of program implementation

**Fig 2:**
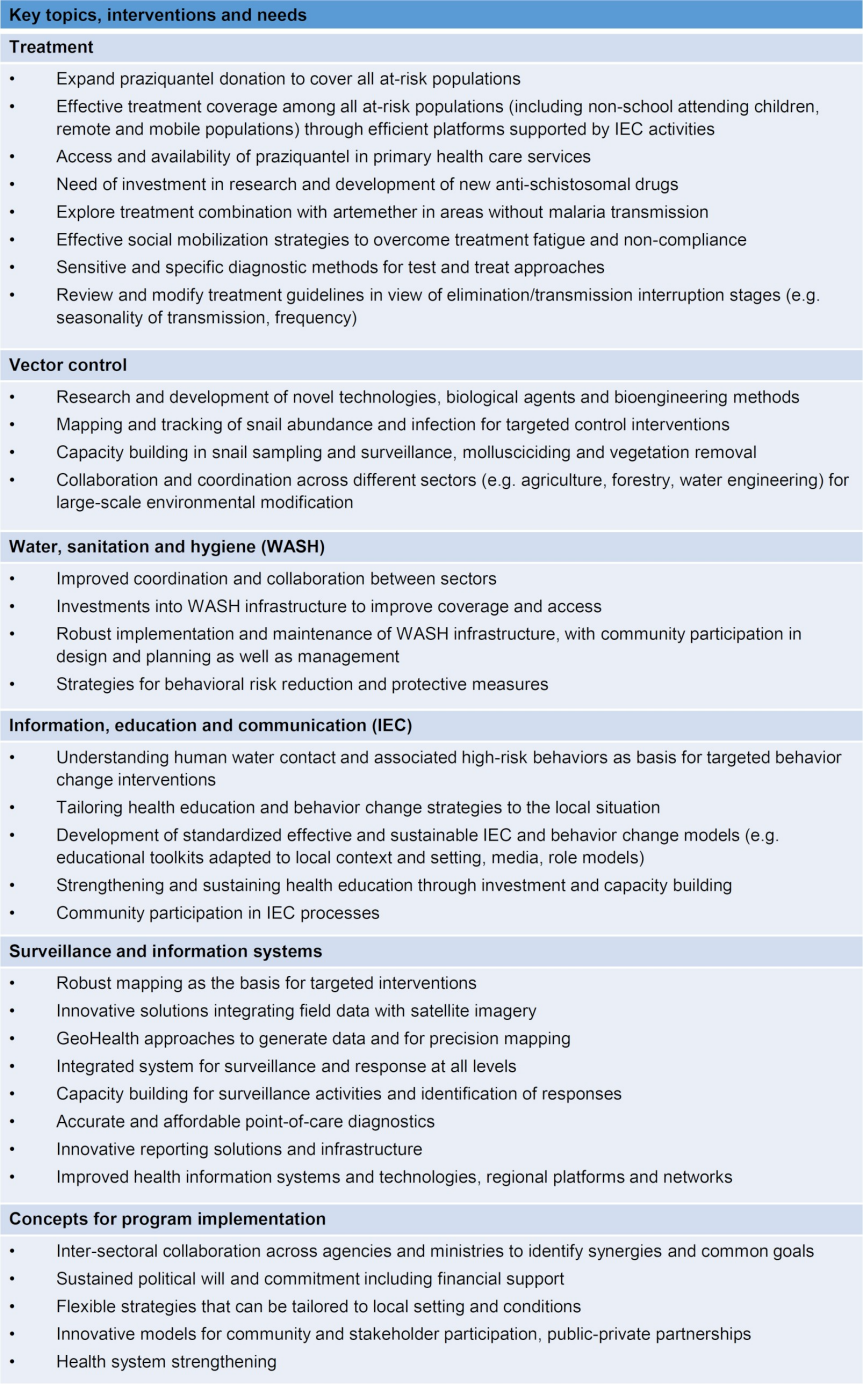
Key topics, interventions and needs for advancing towards breaking schistosomiasis transmission.

The rationale for integrated schistosomiasis control and elimination efforts to advance towards interruption of transmission as opposed to the current approach, mainly relying on MDA for elimination of schistosomiasis as a public health problem, has been discussed repeatedly for non-African settings [11, 21, 24, 36, 38, 55, 63, 90, 115, 116], but also with reference to Nigeria [75], Zanzibar [117] and Egypt [105]. Low acceptance of MDA, vector control or WASH interventions [103, 116] as well as insufficient resources, weak health systems and lack of coordination are a reality in many programs [11, 21, 38, 55, 63, 66, 95, 97, 115, 116]. Impact modeling studies have shown that strategies combining different interventions are more likely to result in successful disease control and elimination [64, 65]. More generally, a human-centered design approach is key for acceptance of interventions, community ownership and successful and sustainable implementation of programs [77]. Fig 2 presents priorities, needs and considerations in addition to identified key topics and interventions from key informants and the reviewed literature.

## Discussion

Solid evidence demonstrates that sustainable control and particularly interruption of transmission and elimination of human schistosomiasis is only possible through multipronged long-term strategies that are adapted to local conditions, sustained by political commitment and funded adequately [22, 27, 63, 74, 92]. Intervention strategies need to be based on (predictive) risk mapping and combine different interventions that are tailored to the local socio-economic and epidemiological context through close consultation with the local population. Costs and other economic factors associated with implementation of interventions represent a critical aspect needed for intervention prioritization and policy change. Targeted cost-effectiveness analyses are valuable tools to guide country programmes and inform strategies and intervention design [118–120]. Mapping must go beyond epidemiological parameters such as disease prevalence and infection intensity in humans. Instead, it must also cover abundance of snails and snail infection rates, transmission sites, access to safe water sources, and human behaviors to guide interventions, as proposed in a systems modelling approach to evaluate and predict effectiveness of interventions [114]. The current focus on school-aged children of the treatment programs is of concern in regards to reaching the set elimination targets. This calls for in-depth analysis on implementation gaps, barriers and needs to establish or improve access to treatment for all at-risk populations. Intervention packages need to cover treatment of infected individuals, snail control in infested water bodies, WASH and human behavior as well as strengthening relevant knowledge, and are ideally implemented by communities and the local health system under the strong leadership of an elimination program also coordinating activities of other sectors. As emphasized by many interviewed key informants and reflected in the literature, understanding perspectives and involvement of the affected communities [53, 77, 101, 121] in the planning of targeted interventions through a bottom-up approach is critical for acceptance and ownership and thus sustainability of most interventions. Engaging communities in vector control activities, treatment campaigns (e.g. through community-directed treatment strategies), behavior change activities, and WASH (such as CL-WASH) has been shown to be feasible, (cost)-effective [121] and sustainable when prioritizing locally appropriate interventions [53, 61, 77, 101, 122–125]. A strong surveillance and response framework ensures quality control and appropriate adaptation of the interventions to changing epidemiological and risk profiles.

Biological, epidemiological, public health and health system considerations suggest a need for a pluralistic governance framework for comprehensive programs targeting human schistosomiasis elimination. Strong global and national partnerships are essential in advocacy for sustained financial and political commitment. This entails the transition from donor-driven programmes to country ownership for sustainability [126]. Based on the successful implementation of such programs in P.R. China and Japan, the following basic tenets have been formulated [74]. Their relevance in terms of success and sustainability has been confirmed by the findings of our literature review and key informant interviews:

1. Local policy development, strong political commitment
2. Use of multiple interventions in integrated fashion through cross-sectoral action
3. Adaptation of interventions for specific eco-epidemiological settings and over time
4. Active participation of the community and other stakeholders (both private and public) underpinned by country ownership
5. Strong linkages to research and learning activities using surveillance and monitoring data

The aim of the landscape analysis was to identify components, concepts and intervention strategies used in past and ongoing parasitic and vector-borne parasitic disease elimination programs in diverse settings that might be applied for advancing interruption of schistosome transmission. Our study did not result in an exhaustive list of potential innovative tools and stand-alone intervention approaches but rather identified crucial components and concepts of strategies to drive progress towards breaking schistosome transmission. The opinions of key informants and evidence from the peer-reviewed literature presented here are largely reflected in the final draft of the new WHO roadmap for NTD control 2021–2030 [8]. All sources call for adequate efforts to address critical gaps and concerted action across sectors to maximize synergies for effective implementation and sustained impact. The proposed strategies and recommendations should be assessed in regards to country perspectives and experiences in the frame of a consultation to inform future implementation research needs and elimination program design. Recognizing the heterogeneity of schistosome transmission settings in terms of ecological and social context, health infrastructure, human capacity and material as well as financial resources, a focused literature review, qualitative research for exploring community perspectives and detailed evaluation of each individual key intervention approach are required. Such intervention-focused reviews can help to identify the range of available options, tools and intervention modalities and their individual strengths and weaknesses before selecting a particular combination of approaches to be included into a strategy for any given setting. Crucially, and as emphasized in many key informant interviews conducted in the frame of this landscape analysis: consistent and high-quality implementation of available tools and interventions, regularly reviewed and pragmatically adapted, is critical to achieve a sustained impact, and can potentially succeed in interruption of schistosome transmission [127].

**Fig 3:**
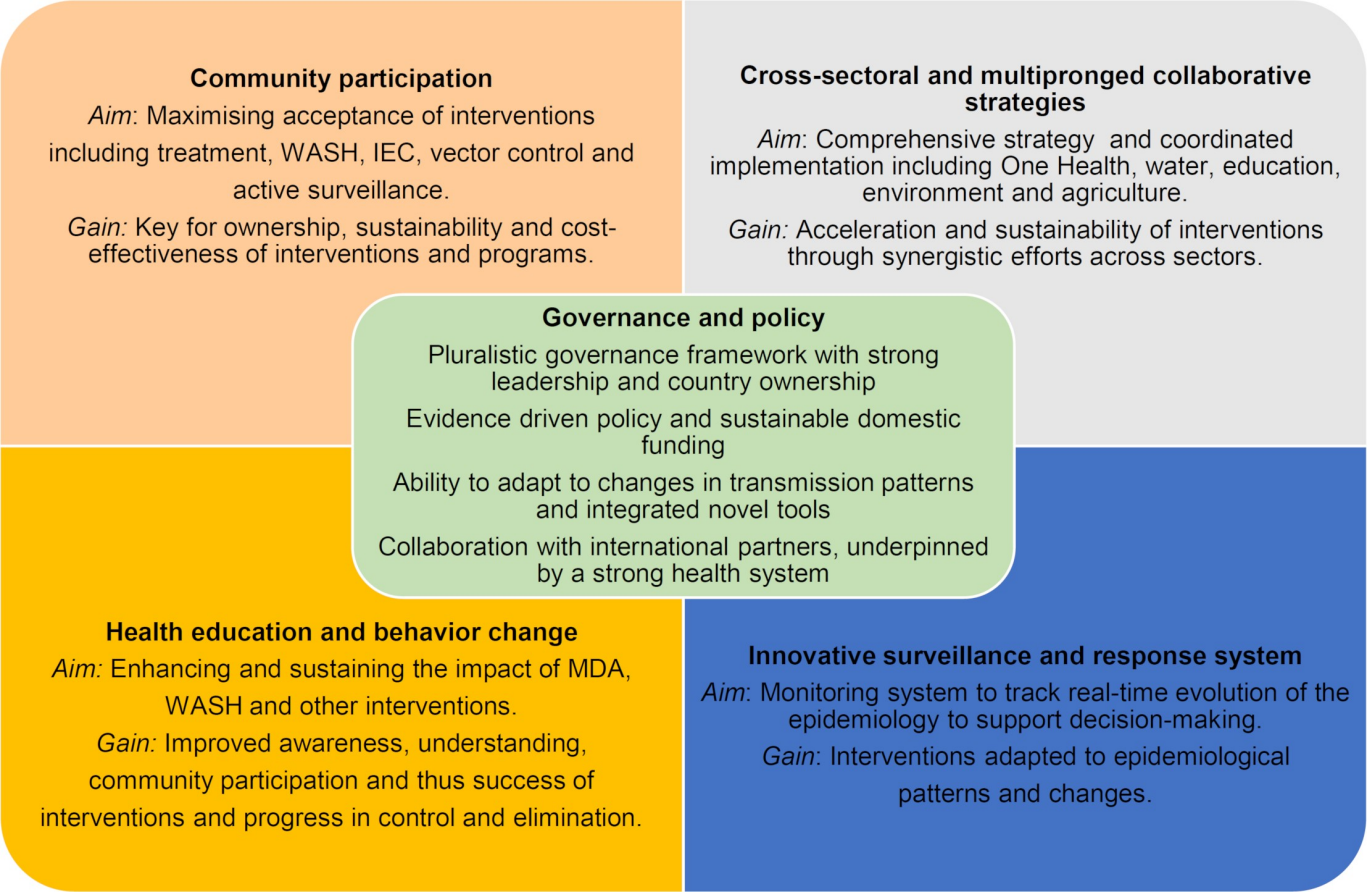
Key components of parasitic disease elimination strategies.

To summarize key messages from our landscape review, five key components, complementing treatment and underpinning integrated comprehensive interventions for parasitic disease elimination, highlight the core dimensions for planning a comprehensive intervention and for defining the concrete approaches that make up an elimination strategy (Fig 3).

### Recommendations and conclusions

**Fig 4:**
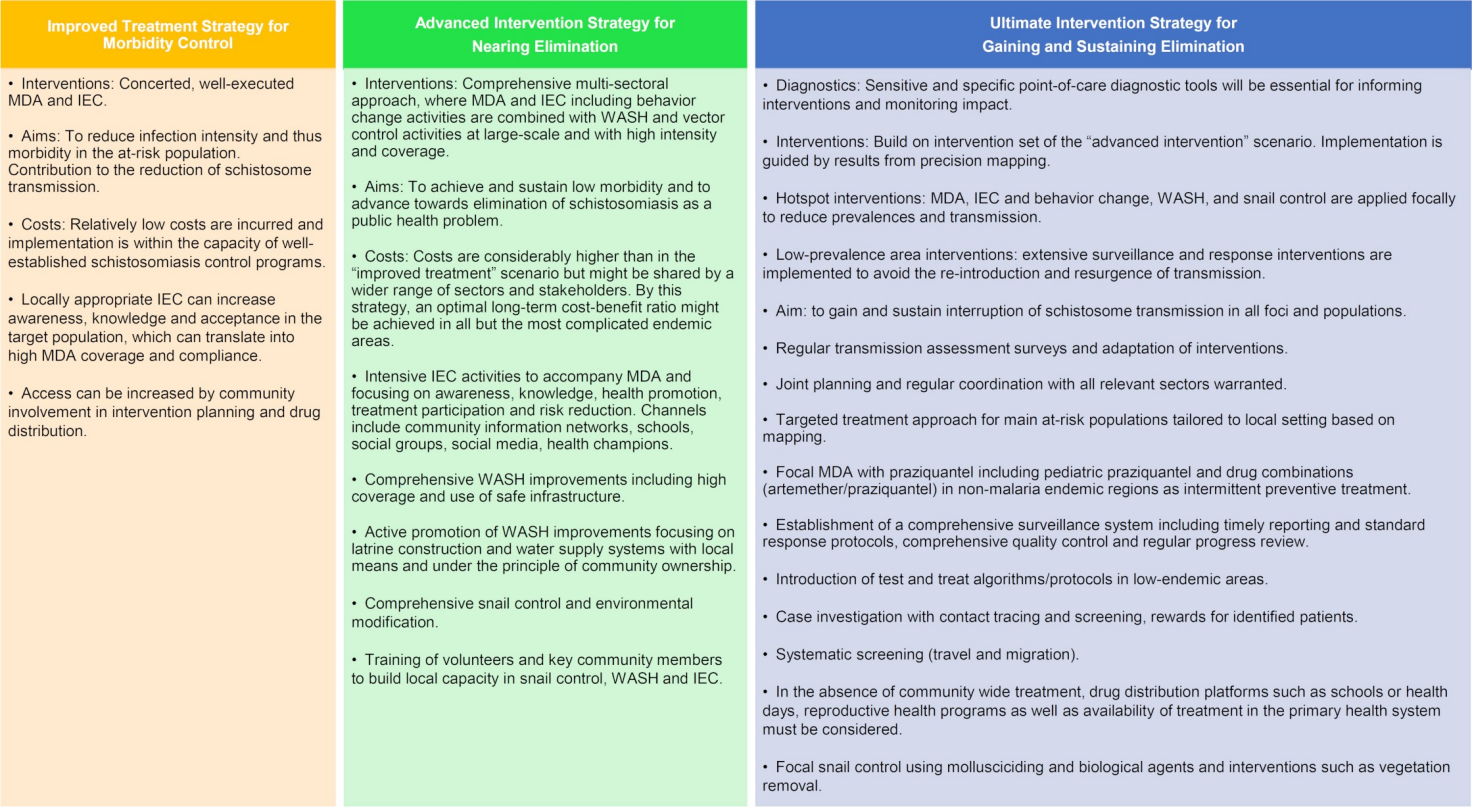
Strategies for strengthening efforts of escalating complexity and ambition towards interruption of schistosome transmission and elimination.

Our landscape analysis reviewed concepts and intervention strategies of past and current parasitic and vector-borne disease elimination programs and identified five key components needed for achieving and sustaining schistosomiasis control and for progressing elimination efforts towards interruption of transmission in sub-Saharan Africa. Based on the evidence from a literature review and information obtained through a series of key informant interviews, we propose three strategies (Fig 4) of escalating complexity and ambition towards interruption of schistosome transmission and elimination, namely i) “Improved treatment strategy for morbidity control”, ii) “Advanced intervention strategy for nearing elimination” and iii) “Ultimate intervention strategy for gaining and sustaining elimination”. For each of the three strategies, we define the aim and list a set of tools and core interventions to address and accommodate the complexity of endemic settings and schistosome transmission dynamics that must be overcome in order to break transmission and advance towards true elimination. Importantly, the decision on the strategy to pursue and the final selection of tools and interventions must be based on a thorough assessment of the local context and take into account resources and timelines.

1. The “Improved treatment strategy for morbidity control” focuses on MDA and IEC. Through locally appropriate IEC, awareness, knowledge and acceptance is increased in the target population, which translates into high MDA coverage and compliance. The strategy aims for a rapid reduction of infection intensity and thus morbidity due to schistosomiasis in the at-risk population. It also contributes to the reduction of schistosome transmission. Relatively low costs are incurred and implementation is within the capacity of well-established schistosomiasis control programs. This strategy is in line with the WHO recommended strategy for morbidity control [7].
2. The “Advanced intervention strategy for nearing elimination” includes a comprehensive multi-sectoral approach, where MDA and IEC including behavior change activities are combined with WASH and vector control activities at large-scale and applied with high intensity. The strategy aims to achieve low morbidity and to advance the impact of programs towards elimination of schistosomiasis as a public health problem. Costs are considerably higher than in the first scenario but might be shared by a wider range of sectors and stakeholders. Relying on this strategy, an optimal medium-term cost-benefit ratio might be achieved in all but the most complicated endemic areas. This strategy is in line with the WHO strategy for elimination and WHA resolution 65.21 [7, 128].
3. The “Ultimate intervention strategy for gaining and sustaining elimination” builds on the set of interventions highlighted under scenario 2. However, MDA, IEC and behavior change, WASH, and snail control are applied focally to areas where the prevalence is above a to-be defined threshold that sustains local transmission. The focal application is guided by the results of extensive precision mapping efforts. In areas where infection levels are below the threshold, extensive surveillance and response efforts will help to avoid the re-introduction and resurgence of transmission and morbidity. Sensitive and specific point-of-care diagnostic tools will be essential for informing interventions and monitoring impact. The aim of the strategy is to gain and sustain interruption of schistosome transmission in all foci and populations, including persistent hotspots and migrant populations. While post-elimination surveillance is suggested by WHO, specific guidance and thresholds on when and where to adapt intervention strategies in near-to-elimination settings is yet to be developed.

## Supporting information

S1 Table Literature review data extraction matrix. (XLSX)

S2 Table Summary of key topics and concepts from the literature review. (DOCX)

S3 Table Thematic analysis. Summary of key topics from key informant interviews.
(DOCX)

S1 Appendix Interview guide. (DOCX)
