# Supplementary material for: Core components, concepts and strategies for parasitic and vector-borne disease elimination with a focus on schistosomiasis: A landscape analysis
Source: PLoS Negl Trop Dis. 2020 Oct 30;14(10):e0008837. doi: 10.1371/journal.pntd.0008837 (PMC7598467; doi:10.1371/journal.pntd.0008837)
Supplement: S2 Table — (DOCX) [file pntd.0008837.s002.docx]

|  | Schistosomiasis (*S. haematobium/ S. mansoni* or *S. japonicum)* | Malaria / Dengue | Onchocerciasis / Lymphatic Filariasis | Other/Vector borne Disease (specify) | Comments |
| --- | --- | --- | --- | --- | --- |
| Treatment   1. Target groups/populations 2. Individual (e.g. test/treat) 3. MDA (stand-alone or combined, focal or general) 4. Algorithm-based 5. Preventive treatment/vaccination 6. Combined treatment | *S. haematobium/S. mansoni*  a. [2-15]  b. [2-6, 16, 17]  c. [2-5, 7, 9, 10, 12-16, 18, 19]  d. [13, 14]  f. [20, 21]  *S. japonicum/S. mekongi:*  a. [3, 9, 13, 22-28]  b. [3, 22, 24, 28-30]  c. [3, 9, 13, 23, 25, 26, 28, 30-36]  d. [13, 24]  e. [25, 26]  f. [20, 28] | malaria:  a. [37-42]  b. [38-46]  c. [37, 38, 45, 47-49]  d. [39]  f. [48] | LF :  a. [50]  c. [21, 50-54]  d.  f. [50, 55]  onchocerciasis:  c. [21, 51, 52, 56, 57]  f. [54] | NTDs :  c. [21, 51, 58-60]  f. [21]  polio, smallpox:  e. [51]  *L. loa* :  c. [52]  STH:  a. [7]  c. [7]  HAT:  b. [56]  c. [53] (cattle) | Malaria :  a.b.c. [38] MDA vs FDA in asymptomatic carrier population  c. [48]: high coverage needed  c. [47] low prevalence consideration  SCH:  a. [11] limited treatment with Fuadin (toxicity)  a.b.c.: [5] repeated MDA without interruption in transmission  f. [20, 28] artemether-PZQ combination therapy, split-dose  c. [25, 30, 31]: PZQ for bovines [18] Astiban and Ambilhar: human, vector, water treatment: pilot study  NTD :  d. [21] : SCH/STH/LF  LF/onchocerciasis/*L. loa*:  d. [52]  f. [52] appropriate treatment strategy for safe combinations |
| Vector Control   1. Focal 2. Large-scale (agricultural practices, environmental modification, ecological, engineering) | *S. haematobium/S. mansoni*  a. [2-4, 9, 11, 13-16, 18]  b. [3, 11, 13, 14, 61]  *S. japonicum/S. mekongi:*  a. [3, 9, 13, 23-25, 27, 28, 30, 31, 33-36, 62-66]  b. [3, 13, 23-25, 30-36, 62-66] | malaria :  a. [39, 40, 43, 47, 49, 53, 67]  b. [47, 53, 67]  dengue :  a. [68-70] | LF :  a. [51-53, 55]  onchocerciasis :  a. [53, 56, 57]  b. [21, 53, 56, 57] | NTDs/VBD:  a. [59, 60, 71, 72]  b. [59, 60, 72, 73]  LF, malaria, dengue:  a. [74]  b. [51, 74]  Guinea worm :  a. [51, 56, 75, 76]  HAT :  a. [53, 56] | *S. japonicum*  [24] modeling combined traditional and ecological approaches  *S. haematobium/*  *S. mansoni:*  [18] Astiban treatment |
| Information – Education – Communication (IEC)  Behavior change  Individual protective equipment | *S. haematobium/S. mansoni*  [2, 3, 7, 10-16, 77]  *S. japonicum/S. mekongi:*  [3, 13, 23-26, 28, 30-33, 35, 36, 78, 79] | malaria:  [37, 39-41, 43, 47, 67, 80]  dengue:  [68, 70] |  | STH/SCH/NTD:  [7, 58-60, 81, 82]  Guinea worm:  [51, 56, 75, 76]  NTD/VBD/ID:  [53, 83, 84] | SCH:  [12] by Community health care providers |
| Access to safe water  Sanitation and hygiene  (WASH: generic, specific) | *S. haematobium/S. mansoni*  [2, 3, 7, 10, 11, 13-16]  *S. japonicum/S. mekongi:*  [3, 13, 23-26, 28, 30-33, 36, 62, 64, 65] | dengue:  [70] | LF:  [55]  NTD:  [73] | STH/SCH/NTD:  [7, 58-60, 81, 82]  Guinea worm:  [56, 75] | STH/SCH/NTD:  [82] personal hygiene higher impact than chemotherapy (for protozoa, hookworm) |
| Surveillance, information system   1. Mapping / TAS 2. Routine surveillance (incl. M&E) (DHIS, notifications etc.) 3. Active surveillance and response incl. contact tracing 4. Zonal, regional (cross-border) collaboration and information exchange | *S. haematobium/S. mansoni*  a. [2-4, 6-8, 11, 13, 14, 16, 85-88]  b. [2, 3, 11, 13, 14, 16, 17, 88]  c. [2-4, 13, 16, 17, 87, 88]  d. [14]  *S. japonicum/S. mekongi:*  a. [3, 13, 22-25, 27-29, 79, 85-91]  b. [3, 13, 22-24, 26, 28, 29, 32, 87, 88, 91, 92]  c. [3, 13, 22-26, 29, 88, 92]  d. [23, 66, 92, 93] | malaria:  a. [38, 40, 45, 67, 88, 94-97]  b. [38, 40, 41, 43, 46, 49, 88, 94, 98, 99]  c. [38, 41-44, 46, 47, 88, 94, 96, 98, 100, 101]  d. [94, 97, 102]  dengue:  a. [68, 69] | LF:  a. [50, 54, 103, 104]  b. [103, 104]  c. [103]  onchocerciasis:  a. [57]  c. [57] | NTDs/VBD/IDs:  a. [50, 52, 53, 58, 71, 72, 84, 86, 88, 105]  b. [53, 71, 72, 84, 88, 105, 106]  c. [53, 71, 84, 88, 105]  d. [88, 93]  LF, malaria, dengue:  c. [74]  d. [51]  polio,smallpox, measles:  b. [51]  Guinea worm:  a. [56, 75, 76]  b. [51, 75, 76]  c. [51, 56, 75, 76]  d. [75] needs  HIV:  a. [96]  c. [96]  STH:  a.[7] | SCH:  a.[85]: systems epidemiology  Guinea worm:  b.[76] Cash rewards  LF/onchocerciasis/*L. loa*:  a. [52]: mapping for appropriate treatment strategy  NTD:  b.[106] integrated NTD surveillance, multiplex assay; sentinel targets for post-MDA surveillance |
| Implementation Strategy:   1. Health system integration 2. Multi-sectoral/multipronged (VC, IEC, WASH, PC, surveillance) 3. Community-action (VC: community volunteers, sensitization/mobilisation, participation) 4. Disease integration (structure, One Health, Systems Epidemiology) | *S. haematobium/S. mansoni*  a. [2, 3, 16]  b. [2-4, 9-11, 13-16, 85]  c. [3, 13, 77]  d. [85]  *S. japonicum/S. mekongi:*  a. [3, 66]  b. [3, 9, 13, 23, 24, 26-28, 30-33, 35, 36, 58, 62-66, 78, 85, 88, 107]  c*.* [3, 13, 26, 28, 78]  d. [85] | malaria:  a. [46, 80]  b. [38, 39, 43, 44, 49, 67, 80, 96]  c. [37, 46, 49, 67, 96]  dengue:  b. [68-70]  c. [68-70] | LF :  a. [103]  b. [55]  c. [104]  d. [50, 55]  onchocerciasis :  b. [56, 57]  c. [53, 56, 57]  d. [56] | NTDs/VBDs :  a. [71, 73]  b. [51, 53, 58-60, 72, 73, 81, 82, 84, 88, 108, 109]  c. [53, 58, 59, 73, 82, 84, 108]  d. [21, 53, 58, 59, 108]  LF, malaria, dengue:  b.[74]  c.[74]  d. [51]  Guinea worm :  a. [75, 76]  b. [56, 75, 76]  c. [53, 56, 75, 76]  d. [76]  VBD :  a. [83]  c. [83]  HIV :  b.[96]  c. [96] | LF :  d. [55] dengue, malaria  SCH:  b.[32] emphasis on setting-specific control  NTD :  d. [21] : SCH/STH/LF  [73] : drug administration, supplements  LF/malaria:  d. [51] LLIN synergistic effects  Guinea worm:  d.[76]: HCW from immunization, malaria for surveillance  onchocerciasis:  [56] CDTi |
| Implementation platform (PHC, fixed points, immunization/ child health, ANC, schools) | *S. haematobium/S. mansoni* | malaria:  [39, 80]  dengue:  [70] |  | NTDs:  [73]  Guinea worm:  [76] |  |
| Human and infrastructure capacity   1. Human (health staff, others) 2. Infrastructure | *S. haematobium/S. mansoni*  a. [4, 10, 13]  b. [10, 14]  *S. japonicum/S. mekongi:*  a. [13, 23, 28]  b. [23] | malaria:  a. [37, 46, 49, 67, 80]  dengue:  a. [69, 70] | LF:  a.[52, 104] | Guinea worm:  a. [75], [76]  LF, malaria, dengue:  a. [74]  NTD/VBD :  a.[53, 59, 88]  b. [88] | LF/onchocerciasis/*L. loa*:  a. [52]: CDTi classification of status for treatment guidance |
| Impact   1. Reduction disease prevalence (morbidity, mortality) transmission interruption 2. Vector/habitat reduction 3. Increase in coverage 4. Acceptance (and compliance) 5. Feasibility 6. Sustainability 7. Cost-effectiveness 8. Spin-offs 9. awareness | *S. haematobium/S. mansoni*  a. [2, 9-11]  b. [11, 16]  g. [11]  *S. japonicum/S. mekongi:*  a. [9, 23, 26, 30, 33, 34, 62, 65, 79]  b. [33, 34, 65]  d. [78]  e. [26] | malaria:  b. [67]  d. [67]  e. [67]  f. [41]  dengue:  b. [70]  g. [70]  i. [41] | LF:  feasible blood sample collection in remote dispensaries  e. [104]: vector sample collection  g. [104]  onchocerciasis:  a.[57] | Guinea worm:  a. [75, 76]  STH:  a.[7] | SCH:  a.[9]: Combined interventions and CWT higher reduction than SBT MDA alone  g. [11] biological and environmental control |

1. Al Abaidani, I., et al., *Decline in transmission of schistosomiasis mansoni in Oman.* Infect Dis Poverty, 2016. **5**.

2. Bergquist, R., et al., *Elimination of schistosomiasis: the tools required.* Infect Dis Poverty, 2017. **6**(1): p. 158.

3. de Noya, B.A., et al., *New approaches for the control and eradication of schistosomiasis in Venezuela.* Mem Inst Oswaldo Cruz, 1992. **87 Suppl 4**: p. 227-31.

4. Elmorshedy, H., et al., *Can human schistosomiasis mansoni control be sustained in high-risk transmission foci in Egypt?* Parasit Vectors, 2015. **8**: p. 372.

5. Gazzinelli, A., et al., *Schistosoma mansoni reinfection: Analysis of risk factors by classification and regression tree (CART) modeling.* PLoS One, 2017. **12**(8).

6. Knopp, S., et al., *From morbidity control to transmission control: time to change tactics against helminths on Unguja Island, Zanzibar.* Acta Trop, 2013. **128**(2): p. 412-22.

7. Korir, H.K., et al., *Young Adults in Endemic Areas: An Untreated Group in Need of School-Based Preventive Chemotherapy for Schistosomiasis Control and Elimination.* Trop Med Infect Dis, 2018. **3**(3).

8. Lo, N.C., et al., *Impact and cost-effectiveness of snail control to achieve disease control targets for schistosomiasis.* Proc Natl Acad Sci U S A, 2018. **115**(4): p. E584-e591.

9. Ndao, B., et al., *[Can we overcome schistosomiasis? A Senegalese example].* Bull Soc Pathol Exot, 2015. **108**(1): p. 17-20.

10. Negron-Aponte, H. and W.R. Jobin, *Schistosomiasis control in Puerto Rico: twenty-five years of operational experience.* Am J Trop Med Hyg, 1979. **28**(3): p. 515-25.

11. Noriode, R.M., et al., *Urinary schistosomiasis in school aged children of two rural endemic communities in Edo State, Nigeria.* J Infect Public Health, 2018. **11**(3): p. 384-388.

12. Rollinson, D., et al., *Time to set the agenda for schistosomiasis elimination.* Acta Trop, 2013. **128**(2): p. 423-440.

13. Tchuem Tchuente, L.A., et al., *Moving from control to elimination of schistosomiasis in sub-Saharan Africa: time to change and adapt strategies.* Infect Dis Poverty, 2017. **6**(1): p. 42.

14. Zhang, H., P. Harvim, and P. Georgescu, *PREVENTING THE SPREAD OF SCHISTOSOMIASIS IN GHANA: POSSIBLE OUTCOMES OF INTEGRATED OPTIMAL CONTROL STRATEGIES.* Journal of Biological Systems, 2017. **25**(4): p. 625-655.

15. Alarcon de Noya, B., et al., *The last fifteen years of schistosomiasis in Venezuela: features and evolution.* Mem Inst Oswaldo Cruz, 1999. **94**(2): p. 139-46.

16. Balahbib, A., et al., *Selecting accurate post-elimination monitoring tools to prevent reemergence of urogenital schistosomiasis in Morocco: a pilot study.* Infect Dis Poverty, 2017. **6**(1): p. 75.

17. Sherif, A.F., *A new trend for controlling schistosomiasis in a hyperendemic area at iflaka, U.A. R., by elimination of the parasite from man and vector.* J Egypt Public Health Assoc, 1968. **43**(1): p. 30-76.

18. Siqueira, L.D., et al., *Schistosomiasis: Drugs used and treatment strategies.* Acta Trop, 2017. **176**: p. 179-187.

19. Bergquist, R. and H. Elmorshedy, *Artemether and Praziquantel: Origin, Mode of Action, Impact, and Suggested Application for Effective Control of Human Schistosomiasis.* Trop Med Infect Dis, 2018. **3**(4).

20. Fenwick, A., *Host-parasite relations and implications for control.* Adv Parasitol, 2009. **68**: p. 247-61.

21. Carlton, E.J., et al., *Evaluation of Mammalian and Intermediate Host Surveillance Methods for Detecting Schistosomiasis Reemergence in Southwest China.* PLoS Negl Trop Dis, 2011. **5**(3).

22. Chen, J., et al., *"Farewell to the God of Plague": The Importance of Political Commitment Towards the Elimination of Schistosomiasis.* Trop Med Infect Dis, 2018. **3**(4).

23. Dong, Y., et al., *Role of ecological approaches to eliminating schistosomiasis in Eryuan County evaluated by system modelling.* Infect Dis Poverty, 2018. **7**(1): p. 129.

24. Gordon, C.A., et al., *Asian Schistosomiasis: Current Status and Prospects for Control Leading to Elimination.* Trop Med Infect Dis, 2019. **4**(1).

25. Khieu, V., et al., *Elimination of Schistosomiasis Mekongi from Endemic Areas in Cambodia and the Lao People's Democratic Republic: Current Status and Plans.* Trop Med Infect Dis, 2019. **4**(1).

26. Liang, Y.S., et al., *[Novel strategies and technologies to achieve the transmission control of schistosomiasis in Jiangsu Province].* Zhongguo Xue Xi Chong Bing Fang Zhi Za Zhi, 2012. **24**(2): p. 119-22.

27. Ross, A.G., et al., *A new global strategy for the elimination of schistosomiasis.* Int J Infect Dis, 2017. **54**: p. 130-137.

28. Liang, S., et al., *Surveillance systems for neglected tropical diseases: global lessons from China's evolving schistosomiasis reporting systems, 1949-2014.* Emerg Themes Epidemiol, 2014. **11**: p. 19.

29. Sun, L.P., et al., *A multidisciplinary, integrated approach for the elimination of schistosomiasis: a longitudinal study in a historically hyper-endemic region in the lower reaches of the Yangtze River, China from 2005 to 2014.* Infect Dis Poverty, 2017. **6**(1): p. 56.

30. Qian, C., et al., *Effectiveness of the new integrated strategy to control the transmission of Schistosoma japonicum in China: a systematic review and meta-analysis.* Parasite, 2018. **25**: p. 54.

31. Seto, E.Y., et al., *Toward sustainable and comprehensive control of schistosomiasis in China: lessons from Sichuan.* PLoS Negl Trop Dis, 2011. **5**(10): p. e1372.

32. Wang, X., W. Wang, and P. Wang, *Long-term effectiveness of the integrated schistosomiasis control strategy with emphasis on infectious source control in China: a 10-year evaluation from 2005 to 2014.* Parasitol Res, 2017. **116**(2): p. 521-528.

33. Zhang, S.Q., et al., *Epidemiological Features and Effectiveness of Schistosomiasis Control Programme in Lake and Marshland Region in The People's Republic of China.* Adv Parasitol, 2016. **92**: p. 39-71.

34. Spear, R.C., et al., *The challenge of effective surveillance in moving from low transmission to elimination of schistosomiasis in China.* Int J Parasitol, 2011. **41**(12): p. 1243-7.

35. Zhou, X.N., et al., *Schistosomiasis japonica control and research needs.* Adv Parasitol, 2010. **72**: p. 145-78.

36. Adhikari, B., et al., *Perceptions of asymptomatic malaria infection and their implications for malaria control and elimination in Laos.* PLoS One, 2018. **13**(12): p. e0208912.

37. Bjorkman, A., et al., *Spatial Distribution of Falciparum Malaria Infections in Zanzibar: Implications for Focal Drug Administration Strategies Targeting Asymptomatic Parasite Carriers.* Clin Infect Dis, 2017. **64**(9): p. 1236-1243.

38. Agrawal, V.K., *Plasmodium falciparum Containment Strategy.* Med J Armed Forces India, 2008. **64**(1): p. 57-60.

39. Feng, X.Y., et al., *Surveillance and response to drive the national malaria elimination program.* Adv Parasitol, 2014. **86**: p. 81-108.

40. Rossi, G., et al., *Closing in on the Reservoir: Proactive Case Detection in High-Risk Groups as a Strategy to Detect Plasmodium falciparum Asymptomatic Carriers in Cambodia.* Clin Infect Dis, 2018. **66**(10): p. 1610-1617.

41. Sturrock, H.J.W., et al., *Targeting Asymptomatic Malaria Infections: Active Surveillance in Control and Elimination.* PLoS Med, 2013. **10**(6).

42. Cao, J., et al., *Communicating and monitoring surveillance and response activities for malaria elimination: China's "1-3-7" strategy.* PLoS Med, 2014. **11**(5): p. e1001642.

43. Lu, G., et al., *Challenges in and lessons learned during the implementation of the 1-3-7 malaria surveillance and response strategy in China: a qualitative study.* Infect Dis Poverty, 2016. **5**(1): p. 94.

44. Mosha, J.F., et al., *Epidemiology of subpatent Plasmodium falciparum infection: implications for detection of hotspots with imperfect diagnostics.* Malar J, 2013. **12**.

45. Wang, D., et al., *Application of community-based and integrated strategy to reduce malaria disease burden in southern Tanzania: the study protocol of China-UK-Tanzania pilot project on malaria control.* Infect Dis Poverty, 2019. **8**(1): p. 4.

46. Enayati, A. and J. Hemingway, *Malaria management: past, present, and future.* Annu Rev Entomol, 2010. **55**: p. 569-91.

47. Maude, R.J., et al., *Optimising strategies for Plasmodium falciparum malaria elimination in Cambodia: primaquine, mass drug administration and artemisinin resistance.* PLoS One, 2012. **7**(5): p. e37166.

48. Karunaweera, N.D., G.N. Galappaththy, and D.F. Wirth, *On the road to eliminate malaria in Sri Lanka: lessons from history, challenges, gaps in knowledge and research needs.* Malar J, 2014. **13**: p. 59.

49. Msyamboza, K., et al., *Sentinel surveillance of lymphatic filariasis, schistosomiasis soil transmitted helminths and malaria in rural southern Malawi.* Malawi Med J, 2010. **22**(1): p. 12-4.

50. Hopkins, D.R., *Disease eradication.* N Engl J Med, 2013. **368**(1): p. 54-63.

51. Kelly-Hope, L.A., et al., *A practical approach for scaling up the alternative strategy for the elimination of lymphatic filariasis in Loa loa endemic countries - developing an action plan.* Glob Health Res Policy, 2017. **2**: p. 12.

52. Molyneux, D.H., *Control of human parasitic diseases: Context and overview.* Adv Parasitol, 2006. **61**: p. 1-45.

53. Molyneux, D.H., *Advancing toward the Elimination of Lymphatic Filariasis.* New England Journal of Medicine, 2018. **379**(19): p. 1871-1872.

54. Burkot, T.R., et al., *The argument for integrating vector control with multiple drug administration campaigns to ensure elimination of lymphatic filariasis.* Filaria J, 2006. **5**: p. 10.

55. Molyneux, D.H., *Vector-borne parasitic diseases--an overview of recent changes.* Int J Parasitol, 1998. **28**(6): p. 927-34.

56. Rodriguez-Perez, M.A., et al., *A Roadmap Followed: The Path Towards the Elimination of Onchocerciasis in Latin America.* 2015: p. 155-173.

57. Sato, M.O., et al., *Odds, challenges and new approaches in the control of helminthiasis, an Asian study.* Parasite Epidemiol Control, 2019. **4**: p. e00083.

58. Lustigman, S., et al., *A Research Agenda for Helminth Diseases of Humans: The Problem of Helminthiases.* PLoS Negl Trop Dis, 2012. **6**(4).

59. Xu, J., et al., *Integrated control programmes for schistosomiasis and other helminth infections in P.R. China.* Acta Trop, 2015. **141**(Pt B): p. 332-41.

60. Duval, D., et al., *A novel bacterial pathogen of Biomphalaria glabrata: a potential weapon for schistosomiasis control?* PLoS Negl Trop Dis, 2015. **9**(2): p. e0003489.

61. Sun, L.P., et al., *An integrated environmental improvement of marshlands: impact on control and elimination of schistosomiasis in marshland regions along the Yangtze River, China.* Infect Dis Poverty, 2017. **6**(1): p. 72.

62. Yang, X., et al., *SWOT analysis on snail control measures applied in the national schistosomiasis control programme in the People's Republic of China.* Infect Dis Poverty, 2019. **8**(1): p. 13.

63. Yang, Y., et al., *Integrated Control Strategy of Schistosomiasis in The People's Republic of China: Projects Involving Agriculture, Water Conservancy, Forestry, Sanitation and Environmental Modification.* Adv Parasitol, 2016. **92**: p. 237-68.

64. Spear, R., B. Zhong, and S. Liang, *Low Transmission to Elimination: Rural Development as a Key Determinant of the End-Game Dynamics of Schistosoma japonicum in China.* Trop Med Infect Dis, 2017. **2**(3).

65. Zhu, H., et al., *Policy Support and Resources Mobilization for the National Schistosomiasis Control Programme in The People's Republic of China.* Adv Parasitol, 2016. **92**: p. 341-83.

66. Ingabire, C.M., et al., *Using an intervention mapping approach for planning, implementing and assessing a community-led project towards malaria elimination in the Eastern Province of Rwanda.* Malar J, 2016. **15**(1): p. 594.

67. Abbas, A., et al., *Integrated Strategies for the Control and Prevention of Dengue Vectors with Particular Reference to Aedes aegypti.* Pakistan Veterinary Journal, 2014. **34**(1): p. 1-10.

68. Barbazan, P., et al., *Assessment of a new strategy, based on Aedes aegypti (L.) pupal productivity, for the surveillance and control of dengue transmission in Thailand.* Ann Trop Med Parasitol, 2008. **102**(2): p. 161-71.

69. Bos, R., M. Fevrier, and A.B. Knudsen, *Saint Lucia revisited.* Parasitol Today, 1988. **4**(10): p. 295-8.

70. Bergquist, R., et al., *Surveillance and response: Tools and approaches for the elimination stage of neglected tropical diseases.* Acta Trop, 2015. **141**(Pt B): p. 229-34.

71. Naranjo, D.P., et al., *Vector control programs in Saint Johns County, Florida and Guayas, Ecuador: successes and barriers to integrated vector management.* BMC Public Health, 2014. **14**.

72. Holveck, J.C., et al., *Prevention, control, and elimination of neglected diseases in the Americas: Pathways to integrated, inter-programmatic, inter-sectoral action for health and development.* BMC Public Health, 2007. **7**.

73. Das, P.K., *Community participation in vector borne disease control: facts and fancies.* Ann Soc Belg Med Trop, 1991. **71 Suppl 1**: p. 233-42.

74. Beyene, H.B., et al., *Elimination of Guinea Worm Disease in Ethiopia; Current Status of the Disease's, Eradication Strategies and Challenges to the End Game.* Ethiop Med J, 2017. **55**(Suppl 1): p. 15-31.

75. Hopkins, D.R., et al., *Eradication of dracunculiasis from Pakistan.* Lancet, 1995. **346**(8975): p. 621-4.

76. Schall, V.T., *Health education, public information, and communication in schistosomiasis control in Brazil: a brief retrospective and perspectives.* Mem Inst Oswaldo Cruz, 1995. **90**(2): p. 229-34.

77. Chen, L., et al., *Health Education as an Important Component in the National Schistosomiasis Control Programme in The People's Republic of China.* Adv Parasitol, 2016. **92**: p. 307-39.

78. Guang-Han, H., et al., *[Challenges and strategies of health education and health promotion in stage of schistosomiasis elimination].* Zhongguo Xue Xi Chong Bing Fang Zhi Za Zhi, 2018. **30**(2): p. 117-120.

79. Bruce-Chwatt, L.J., *Malaria and its control: present situation and future prospects.* Annu Rev Public Health, 1987. **8**: p. 75-110.

80. Campbell, S.J., et al., *Tailoring Water, Sanitation, and Hygiene (WASH) Targets for Soil-Transmitted Helminthiasis and Schistosomiasis Control.* Trends Parasitol, 2018. **34**(1): p. 53-63.

81. Prociv, P., *Parasite elimination programs: home and way.* Medical Journal of Australia, 2002. **177**(6): p. 335-336.

82. Gillette, H.P., *Health education of the public: its role in the eradication of vector-borne disease.* Bull World Health Organ, 1963. **29 Suppl**: p. 183-7.

83. Tambo, E., et al., *Strengthening community support, resilience programmes and interventions in infectious diseases of poverty.* East Mediterr Health J, 2018. **24**(6): p. 598-603.

84. Krauth, S.J., et al., *A Call for Systems Epidemiology to Tackle the Complexity of Schistosomiasis, Its Control, and Its Elimination.* Trop Med Infect Dis, 2019. **4**(1).

85. Malone, J.B., et al., *Use of Geospatial Surveillance and Response Systems for Vector-Borne Diseases in the Elimination Phase.* Trop Med Infect Dis, 2019. **4**(1).

86. Tong, Q.B., et al., *A new surveillance and response tool: risk map of infected Oncomelania hupensis detected by Loop-mediated isothermal amplification (LAMP) from pooled samples.* Acta Trop, 2015. **141**(Pt B): p. 170-7.

87. Tambo, E., et al., *Surveillance-response systems: the key to elimination of tropical diseases.* Infect Dis Poverty, 2014. **3**: p. 17.

88. Magalhaes, R.J.S., et al., *Geographical distribution of human Schistosoma japonicum infection in The Philippines: tools to support disease control and further elimination.* Int J Parasitol, 2014. **44**(13): p. 977-984.

89. Malone, J., et al., *SCHISTOSOMIASIS: GEOSPATIAL SURVEILLANCE AND RESPONSE SYSTEMS IN SOUTHEAST ASIA.* 2016. **41**(B8): p. 1409-1411.

90. Yang, K., et al., *Establishing and applying a schistosomiasis early warning index (SEWI) in the lower Yangtze River Region of Jiangsu Province, China.* PLoS One, 2014. **9**(4): p. e94012.

91. Zhang, L.J., et al., *The Establishment and Function of Schistosomiasis Surveillance System Towards Elimination in The People's Republic of China.* Adv Parasitol, 2016. **92**: p. 117-41.

92. Zhou, X.N., et al., *From gap analysis to solution and action: The RNAS(+) model.* Acta Trop, 2015. **141**: p. 146-149.

93. *A research agenda for malaria eradication: monitoring, evaluation, and surveillance.* PLoS Med, 2011. **8**(1): p. e1000400.

94. *malERA: An updated research agenda for characterising the reservoir and measuring transmission in malaria elimination and eradication.* PLoS Med, 2017. **14**(11): p. e1002452.

95. Jacobson, J.O., et al., *Surveillance and response for high-risk populations: what can malaria elimination programmes learn from the experience of HIV?* Malar J, 2017. **16**(1): p. 33.

96. Pindolia, D.K., et al., *Quantifying cross-border movements and migrations for guiding the strategic planning of malaria control and elimination.* Malar J, 2014. **13**: p. 169.

97. Premaratne, R., et al., *Malaria elimination in Sri Lanka: what it would take to reach the goal.* WHO South East Asia J Public Health, 2014. **3**(1): p. 85-89.

98. Samuels, A.M., et al., *Community-based intermittent mass testing and treatment for malaria in an area of high transmission intensity, western Kenya: study design and methodology for a cluster randomized controlled trial.* Malar J, 2017. **16**.

99. Cotter, C., et al., *Piloting a programme tool to evaluate malaria case investigation and reactive case detection activities: results from 3 settings in the Asia Pacific.* Malar J, 2017. **16**(1): p. 347.

100. Searle, K.M., et al., *Evaluation of the operational challenges in implementing reactive screen-and-treat and implications of reactive case detection strategies for malaria elimination in a region of low transmission in southern Zambia.* Malar J, 2016. **15**(1): p. 412.

101. Larson, E., R. Gosling, and R. Abeyasinghe, *Eliminating malaria: following Sri Lanka's lead.* Bmj, 2016. **355**: p. i5517.

102. Budge, P.J., et al., *Ongoing surveillance for lymphatic filariasis in Togo: assessment of alternatives and nationwide reassessment of transmission status.* Am J Trop Med Hyg, 2014. **90**(1): p. 89-95.

103. Pi-Bansa, S., et al., *Implementing a community vector collection strategy using xenomonitoring for the endgame of lymphatic filariasis elimination.* Parasit Vectors, 2018. **11**(1): p. 672.

104. Zhou, X.N., R. Bergquist, and M. Tanner, *Elimination of tropical disease through surveillance and response.* Infect Dis Poverty, 2013. **2**(1): p. 1.

105. Lammie, P.J., et al., *Development of a new platform for neglected tr*

*opical disease surveillance.* Int J Parasitol, 2012. **42**(9): p. 797-800.

106. Michael, E. and S. Madon, *Socio-ecological dynamics and challenges to the governance of Neglected Tropical Disease control.* Infect Dis Poverty, 2017. **6**(1): p. 35.
