# Supplementary material for: Core components, concepts and strategies for parasitic and vector-borne disease elimination with a focus on schistosomiasis: A landscape analysis
Source: PLoS Negl Trop Dis. 2020 Oct 30;14(10):e0008837. doi: 10.1371/journal.pntd.0008837 (PMC7598467; doi:10.1371/journal.pntd.0008837)
Supplement: S3 Table — Summary of key topics from key informant interviews. (DOCX) [file pntd.0008837.s003.docx]

| KII | Treatment   1. Strategy 2. Population | Vector Control  Environmental modification | Information  Education  Communi-cation  (IEC)  Behavior change | Access to safe water  Sanitation and hygiene  (WASH) | Surveillance  (M&E, mapping, reporting, data collection) | Implementation Strategy  (community-based, vertical/horizontal, integration into health services, programs) | Multi-sectoral  Cooperation  Areas of potential | Capacity Building, Training | Key  focus / challenge |
| --- | --- | --- | --- | --- | --- | --- | --- | --- | --- |
| 1 | Entire community, SAC, PSAC, non-schoolers, farmers, migrants | + | Compliance, understanding, awareness, involve children | + | Monitoring and reporting, surveillance | Immunization, ANC, child health days  Community-based program | WASH/SCH/ NTD  Strategic partnerships | Training CDDs, locals for monitoring, surveillance | Community participation in all interventions and planning for coverage (lack of funding for IEC) |
| 2 | artemether/PZQ; children, women washing, fishermen (behavioral, occupational)  Test and treat | focal, small scale chemical control, biological | compliance, acceptance  water contact behavior | + | Long-term national surveillance response: contact tracing and investigation, mapping snails (Google earth, RS) | Horizontal program, merging commonalities | Water authority | WASH | Surveillance, mapping (human, vector)  Lack of sensitive POC-RDT and VC |
| 3 | PSAC, SAC, adults; pregnant women, young women  Test and treat | focal (small scale mollusciciding) | Early childhood education, immunization platform, nurses, doctors, prevent water contact | + | Redefine elimination targets (morbidity-based) | horizontal community-based programs,  integration into health systems, immunization  Integrated strategy WASH, HSS | socioeconomic development | Laboratory skills | Integrated comprehensive strategy and defined targets,  Diagnostic algorithms, scale-up POCT;  WASH: costly |
| 4 | PSAC, non-schoolers, girls, rural areas, migrant workers, refugees, occupation | + | early childhood education (WASH, STH, trachoma)  target behavior, awareness, social, women’s groups | alternative water sources, sanitation BC guidance, targeted interventions, intensified, Appropriateness | M&E, good reliable baseline data, water contact proximity schools: risk mapping: GIS | Health promotion/MDA round  Community-based WASH/BC | WASH/NTD: education, finance, culture, environment water, tourism; water-related diseases/WASH/SCH/agriculture NGO collaboration | + | Community-based/led WASH, BC,  Coordination  Lack of WASH maintenance, implementation BC, mobilization |
| 5 | migrants, farmers, women at reproductive age, young adults (students Kenya)  test and treat and track (low prevalence)  hot spots: identify people and test | Pragmatic approach for focal mollusciciding,  snail, vegetation removal | Awareness, education of CDDs, toolkit | Acceptance, buy-in | Risk areas/forces for transmission precision mapping, refine surveillance, tracking positive cases | Community-based VC, involvement  Targeted interventions | WASH/SCH/ NTD | VC | Local involvement of people , lack of sensitivity mapping tools  Surveillance  WASH: maintenance issues, lack of implementation BC, VC; inter-sectoral collaboration |
| 6 | All at-risk, PSAC, migrants, nomadic, men, occupation | Targeted and guided for hot spots; simple tools: slash and tear approach (environmental) | VC, WASH, water contact prevention, components  Acceptability (VC), sensitization | Changing water contact points | Mapping, water contact studies, standardized surveillance (active/passive), sampling pre-TAS like; tropical data | Integrated interventions at hot spots  Program flexibility  tropical data platform integration (protocols) | WASH/NTD (limited impact for SCH)  Education  Education/SCH |  | Treatment coverage and informing strategy, surveillance  Challenge : PZQ procurement for non-SAC, VC: labor-intense |
| 7 | Treat entire populations, PSAC, adults (compliance)  Test and treat strategy (schools, health facilities) | Community participation | Compliance, acceptance, knowledge, communication | + | Risk mapping, passive monitoring  Surveillance-response integrated into health system | Integrated interventions  Integration into health services with community involved in control and monitoring activities, IEC  Stand-alone program with some combined activities | WASH/ agriculture  VC: SCH/malaria  PSAC/ immunization |  | Community-participation in control activities, surveillance  WASH issues (maintenance, infrastructure), BC |
| 8 | Identify risk population and tailored intervention | Community engagement: spraying (malaria) | understanding | Community engagement | Flexible M&E framework, accurate data  practical mapping tools (GPS); contact tracing | Vertical program with integration into health services in periphery, public-private  Community-based WASH  Identify at-risk pop for mutual activities (HIV) | Malaria: tourism, development  Military and health services; WASH |  | Participatory approach at district level;  operational quality differences Asia-Africa  (malaria) |
| 9 | Targeted approach for hot spots; CBT vs SBT impact, pregnant women, transient populations, target all at-risk | Focal, targeted VC, concrete lining, economized farming (Asia) | Community-engagement (WASH), compliance | Community buy-in, ownership of latrines and uptake (Indonesia) | Prediction mapping, GIS, RS (HWCS, snails), active surveillance-response, integrated platforms | Integrated multicomponent (SAFE, malaria) community-based strategy  protocol: phased algorithm for elimination, tailored  integrate mutual activities on priority diseases/ education + MDA | Economized farming (Asia)  WASH/social science/ education |  | Integrated multicomponent approach, community-based  diagnostic needs for surveillance |
| 10 | Cover entire at-risk population, SAC, migrants, country specific risk groups  test and treat for depending on stages/prevalence | + | + | +  (sanitation less impact on *S. haematobium)* | Mapping (human and snails), surveillance | Multipronged strategy  Integration into health services for CWT, targeted interventions; incorporation drug delivery; WASH | Agriculture: change farming practices; water system, engineering |  | Multipronged strategy  Lack of sensitive diagnostic tools, health education is difficult |
| 11 | Area/setting specific risk groups, behavioral, occupational, mobile pops, PSAC,SAC  targeted approach  test and treat | + | awareness | + | Surveillance system (active and passive), notification, | Integrated interventions; comprehensive guidance for surveillance (TAS-like), thresholds | WASH/SCH  education in health centers/ANC |  | Integrated strategy, collaboration  Lack of diagnostics for surveillance, inter-sectoral collaboration, WASH maintenance |
| 12 | Review treatment and delivery strategy for low prevalence |  | Coverage, understanding,  demand | + | Surveillance system platforms (independent of disease); risk mapping; M&E framework (TAS for LF: guidance, consultation) | Comprehensive flexible development strategy including WASH, education, infrastructural development: coordinated for potential benefits /UHC  community participation | WASH  surveillance platforms |  | M&E framework and surveillance platform  social science component missing  snail monitoring is not cost-effective |
| 13 | Focal: target entire community with high coverage  case finding and management  targeted treatment  PSAC, adults | VC not successful like in onchocerciasis (DDT), LF (ITN) | Habit change for preventing exposure, reduce contact time  awareness | Water tank ownership | Surveillance: active: Intensified and active case finding, sentinel sites; M&E; granular mapping | Integrated interventions: MDA, active surveillance, WASH, BC (VC less practical), integrated into health services and comprehensive care package | WASH/NTDs | Laboratory/health personnel | Surveillance Lack of implementation of recommended interventions  overreliance on MDA |
| 14 | Test and treat,  PSAC, mothers  access to treatment | Target HWCS | Compliance, coverage, awareness,  understanding | Breaking poverty cycle: access to WASH |  | Integrated into routine health services, existing platforms (MCH, others), UHC approach,  community-based control activities | Water ministry (water-borne disease department), WASH/NTD |  | Integration into routine Health system beyond SBT, community-based control activities  lacks full implementation of WASH/NTD |
| 15 | Test and treat  availability of PZQ outside MDA  Morbidity care  non-schoolers, young women, pregnancy, occupational, behavioral  SBT/CWT, DOT (CDDs) | Coordinated VC | Awareness  Human-centered design approach  appropriate education tools  compliance  social groups |  | Precision mapping  Surveillance –response (active case finding, mobile tracking) | Multicomponent integrated strategy,  integration into health services (test, treat, surveillance)  community-based control activities (VC, BC, surveillance | Engineering/ WASH/environmental | VC, monitoring | Multicomponent strategy,  integration into health services  community-based control activities  lack of POC-CAA, inter-sectoral collaboration |
| 16 | Targeted populations  test and treat  migrants, occupational |  | acceptability | + | Mapping at community-level, not only SAC, surveillance | Operational: micro-planning with local capacity, infrastructure, close coordination comprehensive program and tools for stages  Integration into existing activities for access, merging resources, surveillance (Tropical data platform), mapping | Networks with snail control, environment agencies (sectors outside health sector) | + | Surveillance and integrated disease control, micro-planning  lack of sensitive mapping tools, WASH. BC |
| 17 | Targeted  highest coverage  identify risk groups, individuals systematically  migrants, nomadic populations | + | Compliance, coverage, awareness, knowledge and understanding  Acceptance | + | Accurate data, systematic mapping, surveillance-response system (active and routine at all levels) , epidemiological zones | Community-based control activities (MDA, surveillance, VC), supervision, rewards: incorporation into existing programs (i.e. Guinea worm surveillance and polio immunization) | WASH/SCH | + | Coverage and quality data, community-based control, surveillance  needs: sensitive mapping tools, full implementation |
| 18 | Targeted treatment of risk population in low prevalence setting | Targeted control interventions (vegetation removal, snail collection)  VC success where environmental source is present (HAT, Guinea worm, malaria, onchocerciasis) | Compliance, awareness for durable impact | + | Precision spatio-temporal risk mapping (environmental assessment) using RS, satellite imagery, field data etc.); surveillance system (wild life reservoir, reinfection) | Community mobilization and engagement in control activities (VC), integrated strategy (VC, PC, surveillance, etc.) with adequate tools tailored to setting | Agriculture, social science, engineering, water etc. to identify co-benefits | + | Environmental risk assessment for tailored and targeted interventions: multipronged strategy with community engagement |
| 19 | All not in school, PSAC, occupational, migrants?, women at reproductive age (FGS), adult population  morbidity care | Vegetation removal, risk reduction strategy (malaria) | Context, setting specific behavior  Awareness (FGS), treatment uptake, acceptability (PC, WASH)  education doesn’t lead to BC | Alternative water sources: recreational, agriculture, appropriate sanitation | Mapping vector, human, drivers for targeted intervention, surveillance | Community-focused rather than school-based strategy (trachoma), integration into health services (ANC, SRH)  programs: VC, surveillance | WASH/NTD/ education/VC (resource/time saving), environmental agencies  SRH/FGS |  | Complexity of BC: formative research  guidance on WASH/NTD collaboration |
| 20 | Targeted treatment: children, adults, non-schoolers, PSAC, adolescents, adults, population movement  test and treat (depending stage); efficient MDA strategy: i.e. in focal transmission, country specific |  | compliance | Improve WASH | Micro-mapping (LF) for targeted treatment (GeoHealth,), surveillance-response | Integrated multipronged strategy (PC, WASH, VC, IEC, HSS), identify NTD overlap; defined elimination and full implementation, synergies UHC context | WASH/  engineering/BC/water/ mHealth | HCW for diagnosis, health champions | Multipronged strategy , tailored MDA strategy  Needs: Sensitive diagnostics, micro-mapping  unnecessary PC, full implementation |
| 21 | Access everyone; risk-group shift  co-morbidities (screening opportunity), non-schoolers, migrants | Agricultural land use changes | Acceptability, information,  BCC for targeted approach, understanding risks | + | Surveillance (non-standalone), look at zoonotic route of transmission | Integrated package of tailored, targeted interventions, vertical structure embedded in health services, linking with SRH, MCH, immunization, surveillance | WASH/SCH  Establish strategic partnerships  agriculture  environment | Health staff, HCW (have to be convinced: guidelines vs mindlines), health champions, influencer | Social science: Identify risk groups, tailored interventions (malaria risk group changed), challenge: animal |
| 22 | Increase thx Coverage  all at-risk, PSAC, adults, occupational  targeted approach | Restricted use of chemical molluscicides delivery: GPS-drones | Compliance  acceptance  communication | + | M&E tools  Identify sources of transmission, tracking (vectors, human), quality data, micro-mapping, surveillance-response | Integrated tailored interventions (PC, VC, WASH), multi-sectoral coordination  incorporate surveillance-response into health system), immunization, MCH | Water, education, health sector etc. | Technical, front line workers, schools (surveillance, MDA) | Integrated tailored interventions multi-sectoral strategy  Sensitive diagnostics  lacking, production bottleneck |
| 23 | Stage-dependent targeted test and treat strategy:  pre-elimination: entire population; children post-elimination: migrant risk population | Small-scale targeted focal VC vs wide-scale (engineering, agriculture, water projects) | + | Targeted WASH | Surveillance-response system (active/passive) | Phased strategy  Multi-sectoral cooperation and targeted integrated intervention strategy  cross-border-areas (malaria) : one zone one strategy | WASH/education/agriculture/  engineering/NTD (sometimes easier than incorporation with other disease programs) | + (surveillance) | Phased strategy  Multi-sectoral cooperation and integrated intervention strategy  needs: sensitive diagnostics, Defined thresholds for elimination missing |
