# Supplementary material for: Core components, concepts and strategies for parasitic and vector-borne disease elimination with a focus on schistosomiasis: A landscape analysis
Source: PLoS Negl Trop Dis. 2020 Oct 30;14(10):e0008837. doi: 10.1371/journal.pntd.0008837 (PMC7598467; doi:10.1371/journal.pntd.0008837)
Supplement: S1 Appendix — (DOCX) [file pntd.0008837.s004.docx]

Dear ____

My name is Nora Monnier and I work at the Swiss Tropical and Public Health Institute on a project funded by the Bill and Melinda Gates Foundation with the purpose to review strategies and tools for breaking transmission of urogenital schistosomiasis in Zanzibar.

The objective at this stage is to explore persistent challenges and novel ideas in addition to a comprehensive literature review of tools and strategies for vector-borne disease elimination. The aim is to identify and develop potentially relevant and innovative, evidence-based scenarios to be applied in a low prevalence setting, outlined as below.

I will provide you with a brief summary on the background and setting in Zanzibar. The Zanzibar Government and Ministry of Health have acknowledged urogenital schistosomiasis as a priority public health problem and with preventive chemotherapy campaigns, access to clean water and improved sanitation alongside socioeconomic improvements significant progress towards control and elimination of *Schistosoma haematobium* has been achieved over the last decades with overall prevalence in school-age children below 10% in 2012. The strong commitment and implementation of a research project funded by the Bill and Melinda Gates Foundation via the Schistosomiasis Consortium for Operational Research and Evaluation (SCORE) on the islands Pemba and Unguja has further reduced the prevalence and intensity to below 2% and 1% in Zanzibar in 2017 but focal areas of high transmission persist. Currently, bi-annual treatment with Praziquantel in schools and communities take place in selected areas.

Given your experience in important aspects of this field we would welcome the opportunity to learn from your valuable insights. We therefore thank you very much for having accepted to participate in this interview.

All your answers will be treated confidentially and no identifying information will be maintained during analysis; *your name will not be linked to any statement*. *Do you agree that we mention your name as key informant in the report which can be shared upon demand?*

Your participation is voluntary and you can withdraw from the interview at any time with no further consequences.

**Questions**

1. What, do you think, is needed/missing to reach sustained interruption in transmission of (urogenital) schistosomiasis in Zanzibar? *(elimination by WHO: eliminate as PH problem: defined as prevalence <1% of heavy intensity infections in all sentinel sites; interruption of transmission: incidence of infection zero)*
2. In a low prevalence setting
3. In remaining focal high transmission sites

- *Barriers, gaps, challenges and potential solutions*
- *Factors that influence success or failure (commitment, funding, coordination, multi-sectoral, -lateral collaboration, community engagement,…)*

1. What “lessons learnt” or “best practices” from other successful programmes for vector-borne diseases could be applied for schistosomiasis elimination (e.g. Malaria, Onchocerciasis, Trachoma, Dengue, Guinea Worm, LF, others)?
2. Are there any novel promising innovations, tools or strategies that could move towards elimination of schistosomiasis in general?

b) How could existing approaches be optimized?

c) Where would you see the priority for investment, and why?

*(intensified MDA, vector control, behavior change, WASH, surveillance response, TAS, M&E, diagnostics, test and treat…)*

- *feasibility*
- *sustainability*
- *cost-effectiveness*
- *acceptance*
- *fast impact*

1. In order to reach elimination, the entire population needs to be assessed*/targeted*: which are the specific groups at risk (age, gender, occupation, migrant groups) and which groups are being missed? And due to what reasons? How could these groups be targeted? *(policy, surveys, PC, screening, inaccurate diagnostics, treatment coverage)*
2. For a sustainable long-term commitment on the way to elimination

what is/are the role, importance and challenges in regards to:

1. Funding (external vs domestic)
2. Country ownership
3. Political commitment, policy
4. Incorporation into existing control/disease programmes (Malaria, immunization,..) or collaborations with multiple sectors (education, WASH, agriculture,..)

*advance shared goals in regards to cost-effectiveness, funding and resource allocations, resource mobilization*

1. others
2. What are the key elements to be able to react to changing dynamics in transmission patterns?

*Policy changes to guide targeted interventions, diagnostic accuracy, mapping (precision), prediction/impact modelling, surveillance-response system, capacity building, technical expertise and resources, dx*

1. *Optional:* In regards to components of intensified control interventions (e.g. PHASE): what approaches can improve acceptability, effectiveness and sustainability of interventions?
2. Preventive chemotherapy

- *Community engagement/planning, compliance, volunteers , coverage*

1. WASH/access to clean water/Sanitation

- *increase of commodity use*
- *Eliminate dependency on infested local water bodies*

1. IEC and behavioural change interventions

- *How well are BC interventions integrated into control programmes?*
- *Community engagement, social mobilization, communication, resources, social media*

1. Snail control, environmental modification
2. Recommendation for key informants?
